# Keeping the Time: The Impact of External Clock-Speed Manipulation on Time-Based Prospective Memory

**DOI:** 10.5334/joc.388

**Published:** 2024-07-16

**Authors:** Gianvito Laera, Giovanna Mioni, Sandrine Vanneste, Patrizia Silvia Bisiacchi, Alexandra Hering, Matthias Kliegel

**Affiliations:** 1 Cognitive Aging Lab (CAL), Faculty of Psychology and Educational Sciences, University of Geneva, Switzerland; 2 Centre for the Interdisciplinary Study of Gerontology and Vulnerability, University of Geneva, Switzerland; 3 LIVES, Overcoming Vulnerability: Life Course Perspective, Swiss National Centre of Competence in Research, Switzerland; 4 Department of General Psychology, University of Padova, Padova, Italy; 5 UMR CNRS CeRCA 7295 –Universitéde Tours, Tours, France; 6 Padova Neuroscience Center (PNC), University of Padova, Padova, Italy; 7 Department of Developmental Psychology, Tilburg School of Social and Behavioral Sciences, Tilburg University, The Netherlands

**Keywords:** prospective memory, time monitoring, attention, time passage, external clock-speed

## Abstract

Several studies have suggested that time monitoring is important for appropriate time-based prospective memory (TBPM). However, it is still unknown if people actively use internal timing processes to monitor the approaching target time, and whether they do so by tracking the duration between clock digits, or by counting and matching the numerical progression of clock ticks’ digits with the target time. Therefore, in the present study, we investigated whether a manipulation of the external time affected time monitoring and TBPM performance. In two experiments, participants performed two identical TBPM tasks: a first TBPM block with no clock-speed manipulation followed by a second TBPM block, where the clock-speed was manipulated as faster or slower (experimental conditions) or normal (control condition). The results showed that only participants in the slower clock condition increased time monitoring in the second compared to the first TBPM block (*d* = 0.42 and 1.70); moreover, particularly in Experiment 2, participants in the faster clock condition checked the clock significantly less frequently than participants in the slower clock (*d* = –1.70) and in the control condition (*d* = –0.98), but only during the 4^th^ minute. No effect was found for TBPM performance. Overall, results suggested that people tracked the target time by counting and matching the numerical progression of clock ticks’ digits with the target time. The findings are discussed considering the most recent theoretical advancements about the relationship between time perception and TBPM.

Prospective memory is the ability to remember an intention at the appropriate moment in the future (time-based prospective memory: TBPM), or when a particular event approaches (event-based prospective memory: EBPM; Einstein & McDaniel, 1990). In everyday life, an example of TBPM could be to call your boss at ten o’clock, whereas an example of EBPM would be to buy the newspaper when passing the newspaper shop on the way home (for recent meta-analyses, see Laera et al., 2023, and Peper & Ball, 2022). PM is considered highly important for everyday functioning as it is involved in daily activities such as medication management, handling finances, or meal preparation (Haas et al., 2020; Hering et al., 2018; Suchy, 2020; Woods et al., 2015). In the traditional laboratory TBPM task (D. C. Park et al., 1997), people are asked to remember to perform a specific action (e.g., to press Enter every 4 minutes) while they are engaged in a background activity, generally referred to as the ongoing task (OT); people are usually free to check a clock on the computer screen by pressing another key.

There is converging evidence that controlling the clock (i.e., time monitoring) is key for TBPM performance, and several studies have revealed time monitoring being highly correlated to TBPM accuracy (Ceci & Bronfenbrenner, 1985; Gan & Guo, 2019; Harris & Wilkins, 1982; Huang et al., 2014; Mäntylä et al., 2006; Mioni & Stablum, 2014; Vanneste et al., 2016). Moreover, it has been revealed that, beyond the overall frequency of time checks, time monitoring is truly efficient only when deployed strategically. Indeed, several studies have consistently shown that people used the clock in a strategic manner, meaning that they checked the clock few times as the task starts, and then increase the number of clock checks as the PM target time approaches, forming a “J-shaped” curve (Kvavilashvili & Fisher, 2007; Labelle et al., 2009; Mäntylä et al., 2006; Mioni et al., 2017, 2020; Vanneste et al., 2016). This has led to the research question as to which cognitive processes drive time monitoring strategically in a TBPM task (Joly-Burra et al., 2022; Labelle et al., 2009; Lecouvey et al., 2017; Mackinlay et al., 2009; Mioni et al., 2012, 2017, 2020; Vanneste et al., 2016; Waldum & McDaniel, 2016; Waldum & Sahakyan, 2013).

## Cognitive processes of time monitoring

Some authors have argued that TBPM selectively involves a dynamic use of internal time processes, usually described as oscillators, internal “pace-makers” or “internal clock” (Block, 1990; Block & Zakay, 1996; Harris & Wilkins, 1982; Labelle et al., 2009; Lecouvey et al., 2017; Mackinlay et al., 2009; Ogden et al., 2011; Waldum & McDaniel, 2016; Waldum & Sahakyan, 2013; Zakay, 1992; Zakay & Block, 1997). The Test-Wait-Test-Exit model (TWTE) suggests that individuals initially estimate a duration close to the PM target time, periodically updating it by checking the external clock until the critical period, when reliance shifts from internal to external time monitoring by significantly increasing clock-checking (i.e., frequency of test-wait cycles) until the ongoing time matches with the PM target time (Harris & Wilkins, 1982; Mioni & Stablum, 2014). The TWTE model posits that time estimation abilities are crucial for strategic monitoring of the PM target time (Block & Zakay, 1996; Harris & Wilkins, 1982); however, it does not explain the mechanisms through which the internal clock works, or how people compute internal representations of time (Block & Zakay, 2006).

Few experimental studies have explored the involvement of internal time processing in TBPM. For example, Huang et al. (2014) found that reminders prompted participants to check the clock, improving TBPM accuracy, while discouraging clock-checking led to decreased frequency of checks without impairing TBPM performance. The authors concluded that time monitoring in TBPM tasks can be either external and/or internal, driven respectively by the presence of external reminder, and by the possibility/accessibility to clock-checking; the external monitoring of time reflects the maintenance of the intention in mind, whereas the internal monitoring of time allows to track internally the current passage of time (Huang et al., 2014). The role of internal time representation in TBPM was furtherly confirmed by Gan and Guo (2019), who found that training improved both TBPM and time estimation abilities, suggesting that temporal processes involved in both TBPM and time estimation could be shared, at least partially (Gan & Guo, 2019). Besides these few empirical findings, correlational studies supports the positive association between time estimation abilities and time monitoring, especially over the last minute before the PM target time (Mioni et al., 2012, 2020; Mioni & Stablum, 2014; Vanneste et al., 2016). However, correlational findings regarding TBPM performance and temporal abilities are inconsistent: some studies showed that better temporal abilities were positively correlated to higher TBPM accuracy (Mioni et al., 2017; Vanneste et al., 2016), but others did not report such effect (Lecouvey et al., 2017; Mackinlay et al., 2009; Mioni et al., 2012, 2020). Such mixed evidence highlights the necessity of considering multiple dimensions of time perception and its impact on TBPM. For instance, while accurate time estimation may enhance time monitoring behaviors, other cognitive processes, such as attention and memory retrieval strategies, are also important for TBPM performance. Therefore, the present study aimed to delve deeper into these dynamics by manipulating external clock-speed in the TBPM paradigm to assess how changes in time flow influence strategic time monitoring and TBPM performance.

## Temporal and numerical proximity

TBPM involves using external clock time to anticipate the proximity to the PM target time (i.e., how closely the ongoing time is to the PM target time) for executing the intention (Bowden et al., 2017; Smith et al., 2017). Proximity is gauged through temporal and numerical dimensions. *Temporal* proximity refers to constant intervals between the digits displayed on a clock, and it is essential to elaborate an internal representation of time for duration estimation (Jones, 2006; Üstün et al., 2017; Zakay & Block, 2004). In contrast, *numerical* proximity refers to solely the progressive numerical information conveyed by clock digits, and it involves processes of checking and matching the ongoing time with the incoming PM target time via attentional control processes like task-switching (Graf & Grondin, 2006; Peper & Ball, 2022; Strickland et al., 2023).

In the literature, it is often assumed that individuals attend and execute TBPM tasks based on strategic use of internally-generated representation of time driven by the *temporal* proximity between the ongoing clock time and the PM target time, updated at each clock check (Jones, 2006; Üstün et al., 2017; Zakay & Block, 2004). However, other authors argued that monitoring processes in TBPM might be similar to the monitoring processes involved in EBPM and do not require strictly time estimation abilities, but rather attentional control processes (Ball et al., 2015; Graf & Grondin, 2006; Peper & Ball, 2022). Several studies on EBPM supported this, showing improved PM accuracy when the task was supported by contextual information (for a revision, see Peper & Ball, 2022). For example, a study by Bowden and colleagues (2017) showed that, when people are told that the EBPM cue will occur at specific OT trial number intervals, PM accuracy was higher compared to the “standard” condition, in which participants did not received any instruction about the PM cue occurrence. Interestingly, in the “context” condition, participants strategically directed the attentional focus away from the OT towards the trial number in order to detect the PM cue (Bowden et al., 2017; for similar results see Bugg & Ball, 2017; Cona, Arcara, et al., 2015). In these EBPM studies, no temporal information is provided, and therefore monitoring processes involved in EBPM are necessarily on the *numeric* proximity of contextual information (e.g., the numeric progression of trial counter) and, as consequence, do not require strictly time estimation abilities, but rather attentional control processes.

Understanding dimensions of proximity in TBPM is crucial as they raise an intriguing open question: is strategic time monitoring supported by the temporal interval between clock digits (i.e., the *temporal* proximity between ongoing and target time), or simply by the fact that clock includes a progressive numerical set of expected events (i.e., the *numerical* proximity between ongoing and target time)? Investigating this question is not only a matter of theoretical interest, but also holds practical implications for enhancing human prospective memory and time management in daily life: on the one hand, investigating this question can help to advance the comprehension of cognitive processes involved in TBPM (e.g., time estimation and/or attentional processes); on the other hand, it can potentially devise strategies to improve memory and time monitoring in various real-life contexts.

One approach to answer to such question is manipulating the *temporal* proximity between ongoing and PM target times by altering the duration between clock ticks (clock-speed). Studies have shown that faster or slower clock speeds can lead to overestimations or underestimations of time, respectively (Christandl et al., 2018; C. Park et al., 2016; Thönes et al., 2021; for a review, see Thönes et al., 2018). This method starts with a regular clock block followed by an altered clock block (e.g., one second = 800 or 1200 ms). If internal time processes are crucial for TBPM (Gan & Guo, 2019; Huang et al., 2014; Labelle et al., 2009; Mioni et al., 2012, 2020; Mioni & Stablum, 2014; Vanneste et al., 2016) and external time manipulation affects subjective time perception (Christandl et al., 2018; Fetzer & Cristian, 1997; Thönes et al., 2018, 2021), then differences in TBPM performance due to clock-speed manipulation would indicate the role of internal time processing (Block & Zakay, 2006; Fetzer & Cristian, 1997; Harris & Wilkins, 1982; Jones, 2006; Thönes et al., 2018).

### The present study

In the present study, we investigated whether individuals monitor time using *temporal* or *numerical* proximity by manipulating the external clock-speed in the TBPM paradigm, and to assess how it affected strategic time monitoring and TBPM performance. In two experiments, participants performed two identical TBPM blocks: the first block with no clock-speed manipulation, followed by a second block, where the clock-speed was manipulated, and people were randomly assigned either to faster, slower, or control condition. The differences across clock-speed conditions during TBPM blocks allowed to measure the effect of the clock-speed on internal time representation supporting TBPM performance, which should be found only in the second TBPM block and not in the first TBPM block. We measured several behavioral outcomes: the rate of TBPM task completion (in percentage) was calculated as mean proportion of the number of PM tasks accomplished, regardless of the timing of the PM responses; it was used as to measure whether people remembered (or not) to perform the PM task (Bastin & Meulemans, 2002; Yang et al., 2013). The timing error of the PM responses was measured as averaged difference (in seconds) between the time-point when people performed the TBPM task and the time-point required by the TBPM task (positive values indicated later PM responses; negative values indicated earlier PM responses); this measure was used as indicator of the timing error of PM responses (Guo & Huang, 2019). Time monitoring was measured as mean clock check frequency per minute (Labelle et al., 2009; Mioni & Stablum, 2014; Vanneste et al., 2016).^1^

## Experiment 1

Experiment 1 was conducted in the laboratory. Participants performed two identical TBPM blocks: the first TBPM block with no clock-speed manipulation (1 second = 1000 ms) followed by a second TBPM block, where the clock-speed was manipulated in two experimental conditions to which participants were randomly assigned: faster clock (1 second = 800 ms) vs. slower clock (1 second = 1200 ms).^2^ Regardless of the clock-speed condition, the displayed digits of the external clock did not change in both TBPM blocks (i.e., the blocks appeared to have the same duration); instead, they only differed in terms of actual (real) duration.

We predicted that, if participants rely on *temporal* proximity (i.e., the actual time interval between the current time and the PM target time), differences would emerge between the clock-speed conditions in the second TBPM block. Whilst in the first TBPM block (i.e., without clock-speed manipulation) participants’ internal representations of time are updated through monitoring, in the second TBPM block with a faster clock, this internal representation would become inaccurate, causing participants to wait too long to increase time monitoring, thus reducing clock-checking frequency and impairing TBPM performance. Conversely, with a slower clock, the internal time representation would be shorter, making it less likely for participants to miss the critical window, increasing clock-check frequency and improving TBPM performance. This would suggest that internal time processes play a role in TBPM (Block & Zakay, 2006; Harris & Wilkins, 1982). In contrast, if participants rely on *numerical* proximity (i.e., the progressive sequence of clock digits), we predicted no differences between clock-speed conditions: participants would simply count and match the ongoing time with the PM target time based on the clock, regardless of the actual speed. In this case, the attentional resources detecting the PM cue should remain unchanged across conditions. This would suggest that TBPM relies on numerical cues rather than internal time estimation (Graf & Grondin, 2006).

### Methods

#### Participants

This study was powered to detect moderate-to-large differences in behavioral performances between clock-speed conditions (faster vs. slower) over one repeated measure variable (TBPM block: first vs. second); power analysis was carried out using the R-package *WebPower* (Z. Zhang & Yuan, 2018). In the power analysis, we used an effect size of *f* = 0.33 for a mixed ANOVA model (within-between interaction); the power analysis indicated that detecting an effect size *f* of .33, at 80% power (two-tailed α at .05), would require a minimum sample size of 74 participants.^3^ We collected data from 80 participants (age-range: 18–36 years; *M*_age_ = 23; *SD*_age_ = 4.05; 56 females); all of them were recruited using flyers. Six participants (7.5% of the sample size) reported to have a history of neurological or major psychiatric disease within the last 5 weeks (e.g.: epilepsy, depression, anxiety), or to take psychotropic drugs or others affecting the central nervous system. These participants were excluded; moreover, one participant was further excluded because of problems in understanding the TBPM task instructions. Eight participants detected that clock-speed was manipulated (10% of the sample size); 7 belonged to the faster clock condition and 1 to the slower clock condition (see “Supplementary materials” for more information about the experiment design); we further excluded these participants. All the analyses were carried out on a sample of 65 participants (age-range: 18–34 years; *M*_age_ = 23.2; *SD*_age_ = 4.26; 47 females); the number and age of participants in each clock-speed condition are depicted in Table 1. All participants gave their written informed consent before participating in the study that was conducted in accordance with the Declaration of Helsinki, and the protocol had been approved by the ethics commission of the Faculty of Psychology and Educational Sciences of the University of Geneva (PSE.20191004.05); moreover, all of them received monetary compensation of 20 CHF as reimbursement for taking part to the experiment.

**Table 1:** Descriptive statistics of participants’ age and sample size per clock-speed condition.


EXPERIMENTAL CONDITION	EXPERIMENT 1 (LAB.)			EXPERIMENT 2 (ONLINE)		
	
	AGE		*N*	AGE		*N*
	
	*M*	*SD*		*M*	*SD*	

faster	23.4	4.28	27	28.7	4.92	36

slower	23.0	4.30	37	26.4	4.94	39

control				26.4	3.79	39


#### Materials

##### Time-based prospective memory task

Participants performed two identical TBPM blocks on the computer. For both blocks, the TBPM task was to remember to press the ENTER key on the keyboard every 4 minutes; in total, five PM responses were collected for each block; during the first TBPM block, clock-speed was not manipulated (1 second = 1000 ms), whereas in the second TBPM block, clock-speed was manipulated, and participant were assigned randomly to the faster or slower clock condition (between-subject manipulation). For the faster clock, each minute lasted 48 seconds (1 second = 800 ms), whilst for the slower clock, each minute lasted 72 seconds (1 second = 1200 ms). We chose these specific durations of clock’s seconds because previous studies showed that, using these clock-speeds, a negligible portion of the sample should recognized the manipulation of the external time as such (Thönes et al., 2021). In both the first and the second TBPM blocks, participants were free to check the clock as often as they wanted by pressing the SPACEBAR; if they did so, a digital clock (format: “00:00”) appeared on the screen for 3 seconds (the duration of each second lasted accordingly to the speed of the clock: 2400 ms in the faster clock condition; 3600 ms in the slower clock condition).

##### Ongoing task

While carrying out the TBPM tasks, participants performed a lexical decision task as OT (Meyer & Schvaneveldt, 1971), which asked them to indicate if a string of letters presented on the screen forms a word or not; the procedure was administered in French. We included two OT tasks blocks without additional delayed intention performed before and after the two TBPM blocks, respectively. These tasks were identical to the OT performed during the TBPM blocks; the first OT served as baseline for the PM cost (Guo et al., 2019; McBride & Flaherty, 2020), whereas the second OT served as control for any collateral fatigue effect related to the clock-speed manipulation. Each OT trial started with a fixation cross (1000 ms) followed by the stimulus (2000 ms) and a subsequent black period screen that lasted randomly between 300 and 1000 ms. All the stimuli (words and non-words) had between 5–8 letters; moreover, we selected 1136 stimuli (568 words) based on their highest scores in terms of accuracy and lowest RTs (i.e.: the easiest to detect) following the rules of Ferrand (Ferrand et al., 2010). We chose to use easily detectable stimuli to ensure that the cognitive load related to the OT was the lowest as possible, in order to ensure that the effect of the clock-speed was free from confound effects related to the difficulty of the OT task; at the same time, the random blank period avoided any temporal regularity related to the OT trials, which has been demonstrated to potentially work as temporal cue supporting time monitoring (Guo & Huang, 2019; Heathcote et al., 2015). All OT stimuli were presented in fully randomized order across all the blocks. The total duration of each TBPM block varied between ~16 and ~25 minutes accordingly to the correspondent clock-speed manipulation. Consequently, the OT trials’ number varied across the blocks (~290 trials in blocks with regular clock; ~235 in blocks with faster clock; ~340 in blocks with slower clock). Regardless of the clock condition, all the TBPM task’s blocks had apparently the same duration (21 minutes), meaning that the displayed clock’s digits did not change among the blocks, while the real task duration did.

##### Follow-up questionnaire

We administered a short follow-up questionnaire related to the time manipulation’s awareness (“During this experiment, you did two blocks where we asked you to press the ENTER key every 4 minutes. Did you notice a difference between the first and second blocks? What did you notice?”). Participants were asked to give binary responses (“yes” or “no”) to this question (and to each of the follow-up questions too); in the case of a “yes”, the subjects were asked to provide a short statement of clarification. We explored the subjective reports at the question in a descriptive fashion to detect participants that noticed the manipulation of clock-speed beyond a mere feeling of time moving faster or slower. This procedure was adopted because the clock-speed manipulation should remain undetected, and its eventual effect should be taken into account in the analyses (Thönes et al., 2018, 2021).

#### Procedure

All the computerized tasks were administered using E-Prime 3 (Psychology Software Tools, Pittsburgh, PA, USA), whereas the questionnaires were administered using LimeSurvey (LimeSurvey Project Team / Carsten Schmitz, 2012); in total, a testing session lasted approximately two hours, and comprised also other measures that are out of the scope of the present paper, such as EBPM tasks and finger tapping; these tasks are not described here, but only mentioned in this section for transparency. In order to control for temporal cues that could affect time monitoring, we removed clocks from the testing room; moreover, we closed windows to remove all the temporal influence provided by the day-night cycle, and we kept only artificial lights in the room during the experiment (Barner et al., 2019; Esposito et al., 2015; Rothen & Meier, 2017). As the participants arrived in the laboratory, the experimenter explained the aim of the study, providing an information sheet and the consent form. Once participants accepted to take part at the study, they filled the sociodemographic questionnaire and the State-Trait Anxiety Inventory (Spielberger et al., 1983), and then performed the practice block of the lexical decision task without additional intention, which was identical to the OT administered subsequently in the TBPM blocks. After the experimenter has ascertained that the participant understood the task (e.g., by correcting participants while doing the task, and by clarifying instructions when unclear), participants were asked to perform a finger-tapping task – i.e., rhythmically tapping on the SPACEBAR in two conditions: free tapping and 1-second tapping (X. Zhang et al., 2021). Before passing to the first TBPM block (without clock-speed manipulation), participants performed a practice block lasting approximately 4 minutes, which allowed them to familiarize with the TBPM task. After the experimenter has ascertained that the participant understood the task, the first block of the TBPM block was administered; when the participants completed it, they performed again the finger tapping task, and then the second TBPM block was administered (faster, slower, or regular/external clock condition). Following on this, participants performed an EBPM task in four blocks: two baseline (i.e., without any added intention) and two EBPM blocks (i.e., adding an intention to perform), administered in alternate way. During the baseline blocks, participants worked on an attention task (i.e., deciding whether a presented letter is a small or a capital letter); during the EBPM blocks, participants were asked to press the SPACEBAR once they see the letters M or U appearing on the screen, while they keep doing the same task performed during the baseline blocks. After doing the EBPM tasks, another finger tapping task was administered, followed by another lexical decision task. Once people completed this last task, the State-Trait Anxiety Inventory was administered a second time, followed by the follow-up questionnaire. At this point, the experiment ended, and participants received the monetary remuneration and were debriefed about the aims and background of the study before they left the laboratory.

### Results

We applied mixed-design ANOVAs with post-hoc *t*-tests corrected using multiple comparisons method for the *p*-values of the comparisons. We focused on two effects of interests, as well as on the respective post-hoc comparisons, regardless of the level of significance:

1. The interaction effect Block * Clock-speed (present in all ANOVAs), as a measure of the effect of clock-speed on the dependent variables.
2. The interaction effect Time * Block * Clock-speed, as a measure of the effect of clock-speed on the strategicness of time monitoring (this effect was present only in the analysis on time monitoring).

For all the analyses, Greenhouse-Geisser correction was used when assumptions of sphericity were not met; moreover, we calculated the effect sizes using partial omega squared values (ω²_p_) for statistical inference, and Cohen’s *d* for post-hoc comparisons. The rejection level for inferring statistical significance was set at *p* < 0.05; *p*-values obtained via post-hoc comparisons were interpreted using a significance level calculated with the number of pairwise comparisons for the effect of interest: for Block * Clock-speed, the number of pairwise comparisons was 4, so the significance threshold would be 0.05/4 = 0.013, while for Time * Block * Clock-speed, the number of pairwise comparisons was 16, so the significance threshold would be 0.05 / 16 = 0.003.

Data pre-processing and figures were carried out in R – version 4.2.1 (R Core Team, 2022). In addition to the frequentist analyses, we also ran Bayesian ANOVAs, which was used to quantify how much the model for the null hypothesis is more likely than the model for the alternative hypothesis. Specifically, in the present study, the alternative hypothesis is that there was a difference in the dependent variable as a function of the clock-speed condition over the second TBPM block. We tested the two interaction effects of interest (i.e., Block * Clock-speed, and Time * Block * Clock-speed) against a Bayesian null model containing all other effects that were not of interest (as well as the effect of Participants); this strategy allowed to test strength of hypotheses for these effects of interest against all others. All analyses were carried out in JASP, version 0.18.3 (JASP Team, 2024). Descriptive statistics are reported in Table 2; data, metadata and the R-code of analyses are reported in Open Science Framework (https://doi.org/10.17605/OSF.IO/ST7C5).

**Table 2:** Descriptive statistics (Experiment 1).


	EXPERIMENTAL CONDITION	RATE OF TBPM TASK COMPLETION (%)		TIMING ERROR OF PM RESPONSES (SECONDS)		MONITORING OVER TIME							
		
		FIRST TBPM BLOCK	SECOND TBPM BLOCK	FIRST TBPM BLOCK	SECOND TBPM BLOCK	FIRST TBPM BLOCK				SECOND TBPM BLOCK			
	
						t1	t2	t3	t4	t1	t2	t3	t4

*M*	faster	95.60	90.40	6.82	9.73	0.45	0.66	0.83	2.24	0.30	0.62	0.83	2.01

	slower	85.80	90.50	2.83	–3.60	0.66	0.97	1.20	2.34	0.76	1.23	1.48	2.92

*SD*	faster	8.47	12.90	15.50	14.60	0.48	0.64	0.54	1.39	0.27	0.75	0.58	1.28

	slower	18.00	12.90	14.30	15.90	0.80	1.48	1.52	1.74	1.77	1.88	1.80	2.22


*Note.* Mean and standard deviation for both the prospective memory task performance and time monitoring at Experiment 1 as a function of clock-speed condition (faster vs. slower) and task’s block (First vs. Second). Time-based prospective memory performance is reported as rate of prospective memory tasks completed (in percentage) and as timing error (i.e., as mean response deviation of the prospective response from the target time, in seconds; maximum accuracy = 0; positive values indicate later prospective memory responses; negative values indicate earlier prospective memory responses). Time monitoring is represented as mean clock check frequency in both time-based prospective memory blocks over time (minute 1 vs. minute 2 v. minute 3 vs. minute 4). TBPM: time-based prospective memory; t1: minute 1; t2: minute 2; t3: minute 3; t4: minute 4.

#### TBPM performance

**Figure 1:**
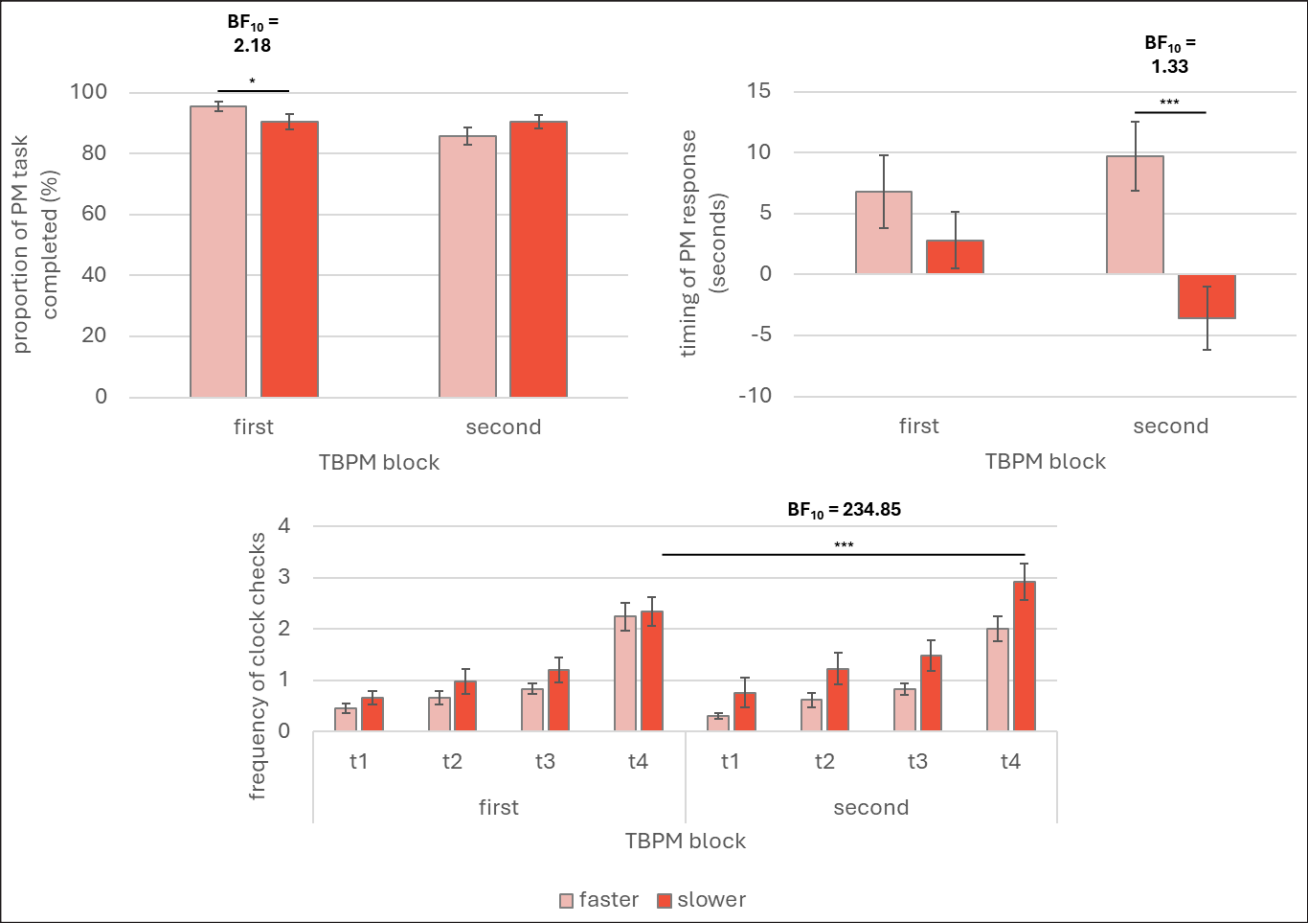
Prospective memory performance and time monitoring (Experiment 1). *Note*. Graphical representations of time-based prospective memory performance and time monitoring from Experiment 1. The upper left panel depicts the prospective memory performance as proportion of completed tasks, regardless of the response’s timing. The right upper panel depicts the timing error of the prospective memory responses, as deviation from the target time (in seconds; maximum accuracy = 0; positive values indicate later prospective memory responses; negative values indicate earlier prospective memory responses). The lower panels depict time monitoring as mean frequency of clock checks over time per time-based prospective memory blocks. PM: prospective memory; TBPM: time-based prospective memory; First TBPM block: prospective memory task without clock-speed manipulation; Second TBPM block: prospective memory task with clock-speed manipulation; t1 = minute 1; t2 = minute 2; t3 = minute 3; t4 = minute 4. Error bars indicate standard error of the means. * *p* < 0.05 ** *p* < 0.01.

Two mixed-design ANOVA were carried out separately for (1) the rate of TBPM task completion – as mean proportion of the number of PM tasks accomplished, regardless of the timing of the PM responses – and (2) the timing error of the PM responses (as difference in seconds between the actual time point when people performed the TBPM task, and the objective time point required by the TBPM task; positive values indicated later PM responses; negative values indicated earlier PM responses). For both analyses, the between-subjects independent variable was Clock-speed (faster vs. slower), whereas the within-subjects independent variable was Block (first TBPM block vs. second TBPM block). The TBPM performance is represented graphically in Figure 1 (upper panels).

The analysis on the rate of TBPM task completion revealed no significant main effect of Block (*p* = 0.922) and Clock-speed (*p* = 0.074), but a significant interaction Block * Clock-speed, *F*(1, 63) = 4.70, *p* = 0.034, ω²_p_ = 0.02. Post-hoc comparisons revealed that such effect was driven mainly by individual differences in the first TBPM block, with participant in the faster clock condition performing better (*M* = 0.96, *SD* = 0.15) than participants in the slower clock condition (*M* = 0.87, *SD* = 0.15), *t*(1213.496) = 2.80, *p* = 0.006, *d* = 0.70, 95% CI [0.01, 1.40]; all other comparisons were not statistically significant (*ps* > .013). Bayesian analysis was carried out testing the alternative model comprising the interaction effect of interest Block * Clock-speed against a null model containing the main effects of Clock-speed, Block and Participants; the Bayes Factor was 2.18, indicating only anecdotal evidence for the alternative hypothesis (Wetzels et al., 2015). The analysis on the timing error of the PM responses did not reveal any significant main effect of Block (*p* = 0.483) as well as interaction effect Block * Clock-speed (*p* = 0.065); however, a main effect of Clock-speed was found, *F*(1, 63) = 9.09, *p* = 0.004, ω²_p_ = 0.06. The difference in the response’s deviations between the faster and slower clock conditions was not found in the first TBPM block, where the mean response’s deviation from the PM target time between faster (*M* = 6.82, *SD* = 15.50) and slower clock condition (*M* = 2.83, *SD* = 14.30) did not differ significantly among each other (*p =* 1); instead, the mean response’s deviation from the PM target time significantly differed between faster (*M* = 9.73, *SD* = 14.60) and slower clock condition (*M* = –3.60, *SD* = 15.90) only during the second TBPM block, *t*(63) = 3.51, *p* < 0.001, *d* = 0.88, 95% CI [0.20, 1.56]. Bayesian analysis was carried out testing the alternative model comprising the interaction effect of interest Block * Clock-speed against a null model containing the main effects of Clock-speed, Block and Participants; the Bayes Factor was 1.33, indicating anecdotal evidence for the alternative hypothesis (Wetzels et al., 2015).

In addition, we ran a series of one-sample *t*-tests to further assess whether the timing of the PM responses in each condition were significantly different from zero, which represented PM responses with no timing error. The analysis on the PM responses during the first TBPM task block for participants exposed later to the faster clock indicated that participants were significantly off-time, *t*(27) = 2.23, *p* = 0.034, *d* = 0.42, 95% CI [0.03, 0.80]; however, the Bayes Factor was 1.66, indicating only anecdotal evidence. The analysis on the PM responses during the second TBPM task block for participants exposed to the faster clock indicated that participants were significantly off-time, *t*(27) = 3.66, *p* = 0.001, *d* = 0.69, 95% CI [0.27, 1.10]; the Bayes Factor was 31.8, indicating very strong evidence. The *t*-test on the PM responses during both TBPM task blocks for participants exposed to the slower clock indicated that participants were on-time (*p* > 0.05).

#### Time monitoring

A mixed-design ANOVA was carried out to measure the effect of Clock-speed (faster vs. slower clock) as between-subject variable, and Block (first TBPM block vs. second TBPM block) and Time (minute 1 vs. minute 2 vs. minute 3 vs. minute 4) as within-subject variables, on time monitoring (measured as mean clock check frequency per minute). Time monitoring is represented graphically in Figure 1 (lower panels). The statistical analysis showed a main effect of the Time, *F*(1.45, 91.29) = 124.15, *p* < 0.001, ω²_p_ = 0.21, as well as interaction effects of Block * Clock-speed, *F*(1, 63) = 7.86, *p* = 0.007, ω²_p_ = 0.01, and Time * Block * Clock-speed, *F*(2.41, 152.02) = 4.15, *p* = 0.012, ω²_p_ < 0.01. Post-hoc analyses for the main effect of Time revealed that people checked the clock strategically overall; specifically, the results showed that participants checked the clock less frequently in minute 1 (*M* = 0.55, *SD* = 1.03) compared to minute 2 (*M* = 0.87, *SD* = 1.37), *t*(63) = –3.18, *p* = 0.002, *d* = –0.23, 95% CI [–0.44, –0.03], minute 3 (*M* = 1.09, *SD* = 1.34), *t*(63) = –5.30, *p* < 0.001, *d* = –0.39, 95% CI [–0.60, –0.17], and minute 4 (*M* = 2.38, *SD* = 1.76), *t*(63) = –17.97, *p* < 0.001, *d* = –1.30, 95% CI [–1.67, –0.93]. Similarly, participants checked the clock less in minute 2 compared to minute 4, *t*(63) = –14.79, *p* < 0.001, *d* = –1.07, 95% CI [–1.40, –0.75]. Clock check frequency was significantly lower in minute 3 than minute 4 too, *t*(63) = –12.69, *p* < 0.001, *d* = –0.92, 95% CI [–1.22, –0.62].

Post-hoc comparisons for the interaction effect Block * Clock-speed showed that people exposed to the slower clock increased significantly clock checks frequency from the first TBPM block (*M* = 1.29, *SD* = 0.03) to the second TBPM block (*M* = 1.60, *SD* = 0.04), *t*(63) = –3.22, *p =* 0.011, *d* = –0.22, 95% CI [–0.40, –0.03]. The same comparisons for participants in the faster clock condition did not show significant results (*p* > 0.013). Post-hoc comparisons for the interaction effect Time * Block * Clock-speed furtherly showed that the interaction effect Block * Clock-speed occurred only during the third and fourth minute before the PM target time; specifically, people exposed to the slower clock increased significantly clock check frequency from the first TBPM block to the second TBPM block, but only on minute 4 (*M_first TBPM block_* = 2.34, *SD_first TBPM block_* = 1.74; *M_second TBPM block_* = 2.92, *SD_second TBPM block_* = 2.22), *t*(63) = –4.88, *p* < 0.001, *d* = –0.42, 95% CI [–0.76, –0.08]. All other comparisons did not show significant results (*p* > 0.003). Bayesian analysis was carried out testing the alternative model comprising the interaction effects of interest (i.e., Block * Clock-speed, and Time * Block * Clock-speed) against a null model containing the main effects of Clock-speed, Block and Participants, as well as the interaction effects of Block * Time, and Time * Clock-speed; the Bayes Factor was 23.46 for the effect Block * Clock-speed, indicating strong evidence for the alternative hypothesis, and >30 for the effect Time * Block * Clock-speed, indicating very strong evidence for the alternative hypothesis (Wetzels et al., 2015).

### Discussion

In Experiment 1, we tested experimentally the effect of internal time processes on time monitoring and TBPM performance by manipulating the external clock-speed. Our results showed that TBPM performance (as both rate of TBPM task completion and timing error of the PM responses) was not affected by the clock-speed (Figure 1, upper panels); however, *t*-tests showed that only participants in the faster clock condition were significantly late during the second TBPM task (*d* = 0.70). Findings on time monitoring showed that people monitored more often when they were exposed to a slower clock, especially over the last minute before the PM target time, in which participants in the faster clock condition checked less than those in the slower clock condition (*d* = 0.42; Figure 1, lower panels).

On the one hand, because both faster and slower clock condition did not differ in terms of both TBPM performance and time monitoring, the results then supported the hypothesis that people “waited” for the PM target time following the numerical metrical events, rather than the temporal relationship between them (i.e., the constant interval between clock ticks); hence, it is likely that participants used the clock not to estimate internally the temporal occurrence of the PM target time, but to detected the *numerical* proximity between the ongoing clock time with the PM target time, regardless of the duration between clock digits. On the other hand, we still found that participants checked the clock more often in the second compared to the first TBPM block, but only when exposed to the slower clock condition, and especially during the last minute before the PM target time; moreover, participants in the faster clock conditions were systematically late during the last minute before the PM target time. Hence, it is possible that the faster clock might have exerted a disruptive effect on internal representations of time that participants might have used to guide monitoring strategically (e.g.: any time representation would be “too long” to keep the pace of the faster clock); in other words, people might have waited “too much time” to reach the critical temporal window around the PM target time (in which it is ideal to increase time monitoring), decreasing in turn the mean clock check frequency (although this effect was not statistically significant, the Bayes Factor was >30, and the effect was present in the pooled sample analyses – see Supplementary materials), and consequently being systematically late at the PM task. On the contrary, slower clock might have exerted a benefit related to slowing down time, meaning that people exposed to the slower clock had objectively more time to elaborate the temporal information. However, compared to the faster clock condition, such advantage is not translated neither into a statistically significant increase of the temporal precision of the TBPM response (i.e., although there was a significant difference between faster and slower clock during the seconds TBPM block, Bayesian analyses indicated no evidence for such difference), nor into a better remembering of the intention itself. Thus, it is likely that these changes reflected merely the fact that participants had more time to complete the task and, and perhaps were expecting the PM target time earlier than the moment of its actual occurrence. However, even though such anticipatory processes were engaged – especially before the PM target time occurrence – it cannot be excluded that internal time processes are involved in TBPM: in this sense, slower clock might have facilitated such anticipatory processes, so people might have used the clock to estimate the temporal occurrence of the PM cue based on the constant duration between hierarchically-organized clock digits (i.e., the *temporal* proximity between the ongoing and the PM target time). In order to further clarify this pattern of results, we replicated and extended the experimental procedure in Experiment 2.

## Experiment 2

In Experiment 2, we aimed to replicate the results obtained from Experiment 1 adding one more between-subjects control condition in which clock-speed was not manipulated (1 second = 1000 ms); such control condition was included to compare both faster and slower clock conditions with a group of participants that were not exposed to any clock-speed manipulation. Experiment 2 was administered online. Overall, the experimental procedure was almost identical between the two experiments, with few minor changes that we made to better adapt the experiment for online testing (see Methods section below).

### Methods

#### Participants

This study was powered to detect moderate-to-large differences in behavioral performances between clock-speed conditions (faster vs. slower vs. control) over one repeated measure variable (TBPM block: first vs. second). In the power analysis, we used an effect size of *f* = 0.33 for a mixed ANOVA model (within-between interaction); the power analysis indicated that detecting an effect size *f* of .33, at 80% power (two-tailed α at .05), would require a minimum sample size of 93 participants. We collected data from 120 participants (age-range: 18–35 years; *M*_age_ = 27.10; *SD*_age_ = 4.65; 64 females); although the power analysis indicated that 93 participants would be sufficient to achieve 80% statistical power, we recruited more participants to account for potential data loss, non-compliance, or unforeseen issues that might render some data unusable. All participants were recruited using Prolific (www.prolific.co), an online platform in which participants receive payment for completion of web-based experiments. We pre-selected healthy participants using the Prolific pre-screening system with the following criteria: age between 18 and 35 years old; being fluent in English; no current alcohol therapy or medication intake, no head injury, long-term health condition/disability, and chronic condition/illness; no mild cognitive impairment/dementia/mental illness. Three participants were excluded because they detected that clock-speed was manipulated (2.6% of the sample size); all of them belonged to the faster clock condition. One participant was excluded because s/he performed the TBPM task each 2 minutes (instead each 4 minutes; presumably s/he did not fully understand the task’s instruction). Two participants (1.8% of the sample size) were furtherly excluded because they pressed the ENTER key at almost every OT trial. All the analyses were carried out on a sample of 115 people (age-range: 18–35 years; *M*_age_ = 27.1; *SD*_age_ = 4.66; 62 females); the number and age of participants in each clock-speed condition are depicted in Table 1 (further power analyses are reported in the Supplementary materials). All participants gave their consent before participating in the study that was conducted in accordance with the Declaration of Helsinki, and the protocol had been approved by the ethics commission of the University of Geneva (CUREG-2022.02.20). Remuneration for the participation was carried out using the Prolific’s system, and it was set at 8 £ per hour. The remuneration was delivered according to the duration taken by each participant to finish the experiment; as the experimental procedure took on average 45 minutes (minimum = 32 minutes, maximum = 102 minutes), people were paid on average 6 £.

#### Materials

##### Time-based prospective memory task

Participants performed identical TBPM tasks administered in the laboratory; the paradigm was almost identical as in Experiment 1, participants performed two TBPM tasks asking them to press the ENTER key on the keyboard every 4 minutes (i.e., within-subject manipulation); during the first TBPM block, clock-speed was not manipulated (1 second = 1000 ms), whereas in the second TBPM block, clock-speed was manipulated, and participants were assigned randomly to three clock-speed conditions (faster vs. slower vs. control condition; between-subject manipulation). As in Experiment 1, for the faster clock, each minute lasted 48 seconds (1 second = 800 ms), whilst for the slower clock, each minute lasted 72 seconds (1 second = 1200 ms). In the control condition, clock-speed was not manipulated (1 second = 1000 ms); in other words, the first and second TBPM blocks were temporally identical.

Although the TBPM paradigm was almost identical between experiments, we reduced the number of PM tasks per TBPM block, to avoid bad data quality from the online assessment. In Experiment 1, we collected five PM responses each block, whereas in Experiment 2, we collected two PM responses for the first TBPM block (i.e., the block *without* clock-speed manipulation), and four PM responses were collected for the second TBPM block (i.e., the block *with* clock-speed manipulation). This was made to limit the overall duration of the procedure and to avoid that participants withdraw due to a long procedure (Finley & Penningroth, 2015; Logie & Maylor, 2009).

##### Ongoing task

While carrying out the TBPM tasks, volunteers performed a lexical decision task as OT (Meyer & Schvaneveldt, 1971).^4^ The OT was in English (diversely from the OT in Experiment 1, which was in French). We chose to switch the language because very few people in Prolific are fluent French-speakers, which in turn decreases the number of potential eligible participants; instead, many participants on Prolific are fluent in English. The OT trial structure was identical to the OT administered in Experiment 1. The OT stimuli were taken from the English Lexicon Project (Balota et al., 2007; Goh et al., 2020), and they were selected following the same criteria used in Experiment 1. We selected in total 596 stimuli (298 words). The total duration of the first TBPM block was ~8 minutes (~135 OT trials), whereas the second TBPM block varied between ~13 and ~19 minutes accordingly to the correspondent clock-speed manipulation. Consequently, the OT trials’ number varied across the blocks (~268 OT trials in blocks with regular clock; ~215 in blocks with faster clock; ~321 in blocks with slower clock).

To prevent participants online from performing the tasks poorly, we included an additional check during the tasks that was not present in Experiment 1: if people did not respond to more than three OT trials in a row, the OT stopped, showing the following message: “It looks like you have stopped to give answers to the requested task. Please resume the task by pressing the ‘p’ key on your keyboard. Thanks for your collaboration.”; once the participants pressed “p”, the OT continued. If people pressed the “p” key more than three times during the tasks, s/he was subsequently excluded from the analysis. This procedure has proved to be effective in eliminating the presence of missing data (0% overall), as only 5 people reported having pressed the “p” key, but no more than once during the whole experimental procedure.

##### Follow-up questionnaire

We administered the same follow-up questionnaire used in Experiment 1. One question was about the time manipulation’s awareness (“During this experiment, you did two blocks where we asked you to press the ENTER key every 4 minutes. Did you notice a difference between the first and second blocks? What did you notice?”).

#### Procedure

The entire experimental procedure has been programmed using Psychopy version 2021.2.3 (Peirce et al., 2019), and hosted on Pavlovia (https://pavlovia.org/; Bridges et al., 2020), which was integrated into Prolific for the experiment’s execution. In total, a testing session lasted approximately 45 minutes. Prior to participation, all relevant information concerning the experimental procedure and data access were provided in written form on the screen; participants provided informed consent to anonymous data usage before participation in the study. If participants accepted to take part in the study, they were introduced to the OT baseline; however, before passing to the practice block, they went through an instruction quiz (i.e., participants had to answer correctly to questions on the task’s instructions before proceeding; Finley & Penningroth, 2015). If participants responded correctly to all the questions of the instruction quiz, they performed a short practice session of the OT baseline, which comprised 8 trials (4 words and 4 non-words). Once participants reached an OT accuracy of at least 80%, the OT baseline was administered; such additional constraint was adopted to further ensure that participants correctly understood the task. When they completed the OT baseline, participants performed the finger tapping task (as described in the procedure of Experiment 1), and then the TBPM task was introduced, with participants answering to a new instruction quiz including the instructions of the TBPM task. As for the OT baseline, if participants responded correctly to all the questions of the instruction check quiz, the practice block was administered, which lasted approximately 4 minutes, allowing the participant to familiarize with the TBPM task. If participants correctly performed the PM response, and reached an OT accuracy of at least 80%, the first TBPM block started. When participants completed it, the second TBPM block was administered (faster, slower, or control condition). Following on this, participants performed another lexical decision task. Once participants completed this last task, the follow-up questionnaire was administered. At this point, the experiment ended, and participants were debriefed about the aims and background of the study; then, they had to provide an a-posteriori consent for the data usage after the experiment’s debriefing, before receiving the remuneration.

### Results

Overall, we applied mixed-design ANOVAs with post-hoc *t*-tests corrected using Bonferroni’s method for the *p*-values of the comparisons (indicated in the text as *p*). As in Experiment 1, we focused on two effects of interests:

1. The interaction effect Block * Clock-speed (present in all ANOVAs), as a measure of the effect of clock-speed on the dependent variables;
2. The interaction effect Time * Block * Clock-speed, as a measure of the effect of clock-speed on the strategicness of time monitoring (this effect was present only in the analysis on time monitoring).

Greenhouse-Geisser correction was used when assumptions of sphericity were not met; moreover, we calculated the effect sizes using ω²_p_ for statistical inference, and Cohen’s *d* for post-hoc comparisons. The rejection level for inferring statistical significance was set at *p* < 0.05; as in Experiment 1, *p*-values obtained via post-hoc comparisons were interpreted using a significance level calculated with the number of pairwise comparisons for the effect of interest: for Block * Clock-speed, the number of pairwise comparisons was 6, so the significance threshold would be 0.05 / 6 = 0.008, while for Time * Block * Clock-speed, the number of pairwise comparisons was 24, so the significance threshold would be 0.05 / 24 = 0.002. Descriptive statistics are reported in Table 3. As in Experiment 1, we also ran Bayesian ANOVAs in addition to the frequentist analyses, which was used to quantify how much model for the null hypothesis is more likely than the model for the alternative hypothesis (Wetzels et al., 2015).

**Table 3:** Descriptive statistics (Experiment 2).


	EXPERIMENTAL CONDITION	PROPORTION OF TBPM TASK COMPLETION		TIMING ERROR OF PM RESPONSES (SECONDS)		MONITORING OVER TIME							
		
		FIRST TBPM BLOCK	SECOND TBPM BLOCK	FIRST TBPM BLOCK	SECOND TBPM BLOCK	FIRST TBPM BLOCK				SECOND TBPM BLOCK			
	
						t1	t2	t3	t4	t1	t2	t3	t4

*M*	faster	95.83	96.53	1.38	4.99	1.08	1.38	1.78	4.07	0.94	1.45	1.84	3.55

	slower	98.72	98.72	2.50	1.60	1.67	2.22	2.12	5.13	1.58	1.99	2.79	6.35

	control	96.15	98.08	2.22	2.60	1.46	1.54	1.92	4.71	1.38	1.80	2.39	5.14

*SD*	faster	14.02	8.77	5.45	11.62	0.80	0.96	1.04	2.19	0.83	0.90	1.12	1.50

	slower	8.01	5.59	3.39	3.50	1.41	2.30	1.19	2.50	1.18	1.29	1.68	2.29

	control	13.50	6.75	1.90	1.83	1.23	1.35	1.35	2.32	1.57	2.01	1.98	2.14


*Note*. Mean and standard deviation for both the prospective memory task performance and time monitoring at Experiment 2 as a function of clock-speed condition (faster vs. slower) and task’s block (First vs. Second). Time-based prospective memory performance is reported as rate of prospective memory tasks completed (in percentage) and as timing error (i.e., as mean response deviation of the prospective response from the target time, in seconds; maximum accuracy = 0; positive values indicate later prospective memory responses; negative values indicate earlier prospective memory responses). Time monitoring is represented as mean clock check frequency in both time-based prospective memory blocks over time (minute 1 vs. minute 2 v. minute 3 vs. minute 4). TBPM: time-based prospective memory; t1: minute 1; t2: minute 2; t3: minute 3; t4: minute 4.

#### TBPM performance

**Figure 2:**
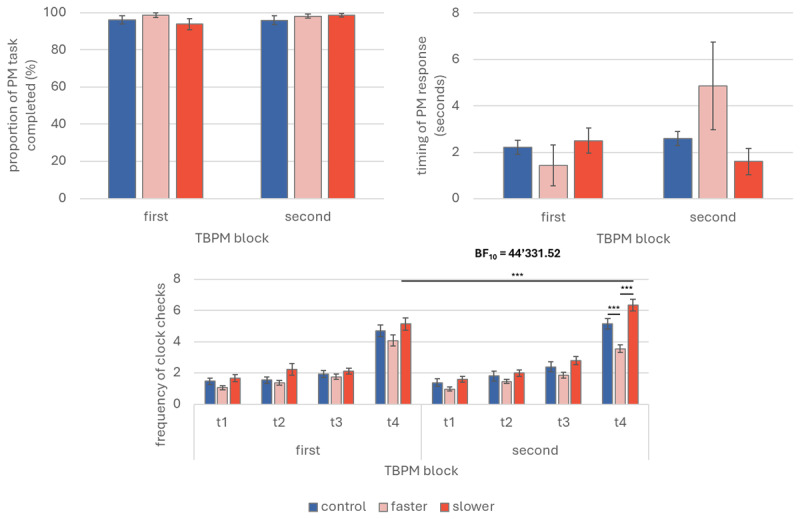
Prospective memory performance and time monitoring (Experiment 2). *Note*. Graphical representations of time-based prospective memory performance and time monitoring from Experiment 2. The upper left panel depicts the prospective memory performance as proportion of completed tasks, regardless of the response’s timing. The right upper panel depicts the timing error of the prospective memory responses, as deviation from the target time (in seconds; maximum accuracy = 0; positive values indicate later prospective memory responses; negative values indicate earlier prospective memory responses). The lower panels depict time monitoring as mean frequency of clock checks over time per time-based prospective memory blocks. PM: prospective memory; TBPM: time-based prospective memory; First TBPM block: prospective memory task without clock-speed manipulation; Second TBPM block: prospective memory task with clock-speed manipulation; t1 = minute 1; t2 = minute 2; t3 = minute 3; t4 = minute 4. * *p* < 0.05 ** *p* < 0.01.

We carried out the same analyses described in Experiment 1. The only difference was the between-subjects independent variable Clock-speed, which in Experiment 2 comprised the additional control condition (faster vs. slower vs. control). TBPM performance is represented graphically in Figure 2 (upper panels). The analysis on the rate of TBPM task completion revealed no significant main effect of Block (*p* = 0.981) and Clock-speed (*p* = 0.164) as well as no significant interaction Block * Clock-speed (*p* = 0.560). Similarly, the analysis on the timing error of the PM responses revealed no significant main effect of Block (*p* = 0.189) and Clock-speed (*p* = 0.477) as well as no significant interaction Block * Clock-speed (*p* = 0.053). Bayesian analysis was carried out testing the alternative model comprising the interaction effect of interest Block * Clock-speed against a null model containing the main effects of Clock-speed, Block and Participants; the Bayes Factor was 0.13 and 1.42 for the rate of TBPM task completion and the timing error of responses, respectively, indicating either anecdotal or no evidence at all for the alternative hypothesis.

As in Experiment 1, we ran one-sample *t*-tests to investigate whether the timing of the PM responses in each condition were significantly different from zero. The analysis on the PM responses during the first TBPM task block for participants exposed later to the faster clock indicated that participants were on-time (*p* = 0.114). The analysis on the PM responses during the second TBPM task block for participants exposed to the faster clock indicated that participants were significantly off-time, *t*(36) = 3.66, *p* = 0.014, *d* = 0.42, 95% CI [0.08, 0.76]; the Bayes Factor was 3.06, indicating moderate evidence. The analysis on the PM responses for participants exposed to the slower clock indicated that participants were systematically off-time during both the first TBPM block, *t*(39) = 4.67, *p* < 0.001, *d* = 0.74, 95% CI [0.38, 1.09] (Bayes Factor = 667), as well as during the second TBPM block, *t*(39) = 2.93, *p* = 0.006, *d* = 0.46, 95% CI [0.38, 0.79] (Bayes Factor = 6.65). Similarly, the analysis on the PM responses for participants in the control condition indicated that participants were systematically off-time during both the first TBPM block, *t*(39) = 7.07, *p* < 0.001, *d* = 1.12, 95% CI [0.72, 1.51] (Bayes Factor > 100), as well as during the second TBPM block, *t*(39) = 8.64, *p* < 0.001, *d* = 1.37, 95% CI [0.93, 1.79] (Bayes Factor > 100).

#### Time monitoring

We carried out the same analyses described in Experiment 1. The only difference was the between-subjects independent variable Clock-speed, which in Experiment 2 comprised the additional control condition (faster vs. slower vs. control). Time monitoring is represented graphically in Figure 2 (lower panels). The statistical analysis showed a main effect of the Time, *F*(1.70, 190.28) = 436.94, *p* < 0.001, ω²_p_ = 0.46, and no significant interaction Block * Clock-speed (*p* = 0.090). Instead, the triple interaction Time * Block * Clock-speed was statistically significant, *F*(5.45, 305.32) = 7.42, *p* < 0.001, ω²_p_ = 0.01. Post-hoc analyses for the main effect of Time revealed that people checked the clock strategically, indicating that people checked the clock less frequently in minute 1 (*M* = 1.34, *SD* = 1.10) compared to minute 2 (*M* = 1.77, *SD* = 1.45), *t*(111) = –3.54, *p* < 0.001, *d* = –0.23, 95% CI [–0.41, –0.05], minute 3 (*M* = 2.12, *SD* = 1.27), *t*(111) = –7.38, *p* < 0.001, *d* = –0.48, 95% CI [–0.67, –0.29], and minute 4 (*M* = 4.85, *SD* = 2.02), *t*(111) = –32.59, *p* < 0.001, *d* = –2.11, 95% CI [–2.53, –1.70]. Similarly, participants checked the clock less in minute 2 compared to minute 3, *t*(111) = –3.83, *p* < 0.001, *d* = –0.25, 95% CI [–0.43, –0.07], and minute 4, *t*(111) = –29.03, *p* < 0.001, *d* = –1.88, 95% CI [–2.26, –1.50]. Clock check frequency was significantly lower in minute 3 than minute 4 too, *t*(111) = –25.20, *p* < 0.001, *d* = –1.63, 95% CI [–1.97, –1.29].

Post-hoc comparisons for the interaction effect Block * Clock-speed showed that, during the second TBPM block, people exposed to the faster clock made less clock checks (*M* = 1.94, *SD* = 1.45) than people exposed to the slower clock (*M* = 3.18, *SD* = 1.46), *t*(111) = –3.68, *p* < 0.001, *d* = –0.75, 95% CI [–1.37, –0.14]. No other paired comparison of interest was statistically significant (*p* > 0.002). Post-hoc analysis for the interaction Time * Block * Clock-speed showed that people exposed to the slower clock significantly increased clock check frequency from the first TBPM block to the second TBPM block, but only on minute 4 (*M_first TBPM block_* = 5.13, *SD_first TBPM block_* = 3.69; *M_second TBPM block_* = 6.35, *SD_second TBPM block_* = 3.18), *t*(111) = –5.55, *p* < 0.001, *d* = –0.74, 95% CI [–1.28, –0.20]. Moreover, only on minute 4 during the second TBPM block, people in the faster clock condition checked the clock less (*M* = 3.55, *SD* = 2.50) than people in the slower clock condition, *t*(111) = –7.47, *p* < 0.001, *d* = –1.70, 95% CI [–2.68, –0.72], as well as than people in the control condition (*M* = 5.14, *SD* = 2.02), *t*(111) = –4.26, *p* < 0.001, *d* = –0.98, 95% CI [–1.89, –0.07]. All other comparisons of interest were not significant (*p* > 0.002). Bayesian analysis was carried out testing the alternative model comprising the interaction effects of interest (i.e., Block * Clock-speed, and Time * Block * Clock-speed) against a null model containing the main effects of Clock-speed, Block and Participants, as well as the interaction effects of Block * Time, and Time * Clock-speed; the Bayes Factor was 0.57 for the effect Block * Clock-speed, indicating no evidence for the alternative hypothesis, and >30 for the effect Time * Block * Clock-speed, indicating very strong evidence for the alternative hypothesis (Wetzels et al., 2015).

### Discussion

In Experiment 2, we aimed to replicate Experiment 1 adding one more control condition, in which clock-speed was not manipulated. The results from Experiment 1 have been partially replicated in Experiment 2: we found that TBPM performance (as both rate of TBPM task completion and timing error of the PM responses) was not affected by the clock-speed, indicating that clock-speed did not change whether people remembered to perform the PM task nor the temporal precision of their responses (Figure 2, upper panels). These results were in line with findings from Experiment 1. Nonetheless, *t*-tests showed that participants performed later PM responses in all clock-speed conditions, while in Experiment 1 this was true only for participants exposed to faster clock. Results on time monitoring were significant for the last minute before the PM target time, showing that people monitored more often when they were exposed to a slower clock across TBPM blocks (*d* = 0.74); this result was consistent across the two experiments. Results from experiment 2 furtherly showed that individuals monitored less often during the second TBPM block when they were exposed to a faster clock compared to those exposed to both the slower clock (*d* = –1.70) and regular clock (*d* = –0.98): diversely from Experiment 1, where the difference between faster and slower clock in time monitoring was present only on a descriptive level (Figure 1, right panel), in Experiment 2 such difference was statistically significant (Figure 2, right panel).

These findings furtherly supported for the idea that individuals tended to anticipate the PM target time based on numerical cues rather than the passage of time itself, suggesting that they likely checked the clock not to internally estimate when the PM target time would occur, but rather to detect whether the ongoing clock time matched with the PM target time, regardless of the time elapsed between clock digits. Nonetheless, similarly with Experiment 1, we still found that participants in the slower clock condition checked the clock more frequently during the second TBPM block, particularly in the last minute before the PM target time. Yet, this advantage did not lead to a significant increase in the precision of the TBPM response or better recall of the intention, compared to both faster clock and control conditions. These results confirmed that, because participants had more time to complete the task, they might have anticipated the PM target time more easily compared to participants exposed to the faster clock. Hence, it is possible that these anticipatory processes involved internal time mechanisms in TBPM, but only when clock was slower, as it might have facilitated the estimation of the temporal occurrence of the PM cue based on the duration between clock digits.

## General discussion

Despite the conceptually important role of time processing in the core TBPM models, it is not established yet whether internal time plays a crucial role in TBPM or not (Gan & Guo, 2019; Graf & Grondin, 2006; Labelle et al., 2009; Mioni & Stablum, 2014). Therefore, in the present study, we investigated how a manipulation of the external clock-speed in the TBPM paradigm affected strategic time monitoring and TBPM performance through the role of internal time processing. In two experiments, participants performed two identical TBPM blocks: the first TBPM block with no clock-speed manipulation, followed by a second TBPM block, where the clock-speed was altered, and people were randomly assigned either to faster, slower, or control condition (the latter administered only in Experiment 2). We predicted that, if people relied on tracking the *temporal* proximity between the ongoing and the PM target time, then differences between clock-speed conditions during the TBPM experimental block should be found in both TBPM performance (as rate of TBPM task completion and timing of the PM responses) and time monitoring; the internal computation of pure temporal information might then confirm experimentally the assumption that there are internal time processes involved in TBPM. In contrast, if people used the digits displayed on the external clock – rather than the temporal interval between them – we expected no differences between clock-speed conditions during the TBPM experimental block. Hence, it can be argued that TBPM with external clocks could involve only attentional and executive – but not internal timing – processes, which are based on the *numerical* proximity between the ongoing clock time and the PM target time (Bowden et al., 2017; Graf & Grondin, 2006).

Overall, our results from both experiments showed that TBPM performance (as both the rate of TBPM task completion and the timing of the PM responses) was not affected by the clock-speed (Figure 1 and 2, upper panels); however, *t*-tests were not consistent across experiments (i.e., in Experiment 1, only participants in the faster clock condition were off-time during the second TBPM block, while in Experiment 2 this was the case for all conditions), highlighting also important differences across laboratory and online assessment (see also pooled sample analyses in Supplementary materials). Results on time monitoring showed that, especially over the last minute before PM target time, people monitored the external clock more often across TBPM blocks when they were exposed to a slower clock (Figure 1 and 2, lower panels). These results supported the hypothesis that individuals may have checked the clock to match ongoing and target times numerically, updating this match with each check: the more the time advances, the higher the probability that the incoming PM cue is occurring soon, the higher the likelihood that people check the clock, performing accurate PM responses (Marsh et al., 2006; Smith, 2003).

### External clock-speed’s effect and internal time processing

The results from both experiments support idea that participants attended and execute TBPM tasks based on the *numerical* proximity between the ongoing clock time and the PM target time, updated at each clock check. Such idea is well in line with the argument that external clock time provides predictable environmental information, akin to trial counters in EBPM (Peper & Ball, 2022). Therefore, it is possible that strategic monitoring in EBPM may be similar to strategic monitoring in TBPM and does not involve directly duration estimation: while in TBPM individuals track the progression of time, in EBPM participants would use contextual information (e.g., given by OT trials, or by a trial counter, which makes the cue more likely to appear soon) to improve PM accuracy (Anderson et al., 2019; Smith, 2003). Indeed, both TBPM and EBPM tasks involve cue-triggered actions, with TBPM relying on clock checks for time tracking. Interestingly, participants checked the clock more often during the second TBPM block, especially with a slower clock, yet this didn’t notably enhance TBPM accuracy and intention recall compared to other conditions. Hence, increased clock-checking may stem from extended task time and anticipation: anticipatory processes, particularly before the PM target time, possibly benefited from the slower clock, aiding time estimation based on *temporal* proximity between ongoing and PM target time. Therefore, it is possible that different clock-speed potentially influenced time monitoring selectively: slower clocks may favor time estimation, while faster ones may promote attention-driven processes like task-switching.

Another interesting aspect that needs to be further investigated would be which kind of time estimation processes are (particularly) important in TBPM. In the time perception literature, time estimation abilities are measured with time production tasks, which require participants to generate a specific time interval from memory, and with time reproduction tasks, which involve producing an interval of time that corresponds to a previously experienced interval. So far it is unknown which kind of process is involved in TBPM. In our study, the manipulation of external clock-speed may primarily have pertained to time production processes, because participants built an initial representation of the PM target time during the first TBPM block with a regular clock, “encoding” its duration, and then using it in the second TBPM block with altered clock speed. However, future studies are needed to replicate these results and to explicitly disentangle the different timing subcomponents of time estimation, production and reproduction in TBPM.

### Attentional and executive processes in time monitoring

In the present study, clock-speed manipulation seemed to not change the amount of attentional resources deployed to detect the PM cue during the TBPM with the altered clock, regardless the clock-speed manipulation, allowing participants to compute and store the same temporal information – via clock-checking – as they did during the TBPM with regular clock. Although further studies are needed to test this hypothesis, the present study supported the hypothesis that people “waited” for the PM target time (as argued by the TWTE model), but following the numerical metrical events, rather than the temporal relationship between them (i.e., the constant interval between clock ticks). In this scenario, time monitoring and TBPM can be explained without considering the existence of internal time processes computing the temporal relationships between external events, but only accounting for attentionally-driven executive processes based on predictable cues (Graf & Grondin, 2006).

Another aspect involving attentional and executive processes concerns the awareness of the clock-speed manipulation. In our study, participants were not aware of the clock-speed manipulation, and the few who detected the manipulation were excluded. We chose to use this approach to follow the literature, as all the few studies that used clock-speed manipulation kept people unaware of such manipulation (Christandl et al., 2018; Thönes et al., 2018, 2021; Yamane & Matsumura, 2015); indeed, there is no study that investigated whether the awareness of clock-speed manipulation affect people’s behavior. In the TBPM context, people might adapt to the new clock-speed if they expect that the clock-speed will change. Thus, knowing that the clock is altered can affect the way people monitor time and eventually the representation of the external elapsing time, which in turn could affect their TBPM performance. For example, if people are aware that clock are slower, they might choose to allocate more attentional resources to the OT than the PM task, because they expect the PM target time later on; thus, it would not be strategic to monitor as the clock-speed was regular because, with slower clock, the PM target time would occur objectively later compared to the TBPM block in which the clock-speed is not manipulated. On the contrary, people aware of faster clock might decide to focus more on the PM task rather than the OT, because the PM target time is expected to come earlier than the PM target time within the TBPM block without clock-speed manipulation. Temporal expectancy is driven by attentional allocation over time (Bolger et al., 2014; Coull et al., 2011; Coull & Nobre, 1998; Nobre et al., 2007), so the need to check the clock could emerge from the attentional resources dedicated to the PM and to the OT which, in turn, affect the internal representation of the elapsing time, as well as how such representation is used strategically to monitor the external time (Jones, 2006; Jones & Boltz, 1989). In this regard, including a further condition of awareness of the manipulation (e.g.: aware vs. unaware), can help to identify to which degree the effect of clock-speed – and the relative temporal expectancies – affect time monitoring and TBPM.

### Limitations and future directions

The present work has some limitations. Firstly, we observed that the residuals of all models showed substantial deviations from the normal distribution; such deviations from normality, coupled with clear evidence of ceiling effects in the PM tasks, suggested that the assumptions underlying our statistical models were not fully met. While our primary analyses were conducted using parametric mixed-design ANOVA, a more robust approach might better account for these deviations and provide more reliable estimates. To facilitate transparency and reproducibility, we have made our data openly available on the Open Science Framework (https://doi.org/10.17605/OSF.IO/ST7C5), encouraging interested researchers to explore alternative analytical strategies and verify our findings through re-analysis. Another limitation is that, in Experiment 1, the difference in the number of completed PM tasks between faster and slower clock conditions at the first TBPM block presented a significant effect. Many explanations are possible, such as random variation inherent in the sample, the sample size and power of the study, as well as individual differences or other uncontrolled variables. Although the experimental design and the analyses were tailored to account for such differences, future studies are needed to furtherly understand this aspect. Finally, a limitation that needs to be addressed by future studies concerns the differences in cognitive demands of temporal perception and cognition (Thönes & Stocker, 2019) at different PM target times (e.g., seconds, minutes, hours, etc.), which is a limitation to the generalizability of the current study: indeed, the two experiments illustrated in this study focused on short laboratory tasks; however, the cognitive processes underlying longer delays might differ from those assessed in this study (Rendell & Thomson, 1999; Rummel et al., 2023). Future research is needed to further elucidate this crucial aspect.

## Conclusions

In two experiments, we found that people exposed to the slower clock showed increased individual mean frequency of clock checks across TBPM blocks. However, we did not find significant differences between experimental (faster and slower clock) and control condition for both time monitoring and TBPM performance. Thus, our results suggested that only attentional and executive processes may be involved in TBPM, at least using the traditional TBPM paradigm, and that participants based their clock-checking strategy on counting and matching the ongoing time with the PM target time (i.e., the *numerical* proximity). This was the first study that introduced a clock-speed manipulation in TBPM; therefore, future studies are needed to replicate the results, and to further understand the role of internal time processing in TBPM.

## Declaration of Generative AI and AI-assisted technologies in the writing process

During the preparation of this work, the author(s) used ChatGPT (OpenAI, 2023) for building the R-script. After using this tool/service, the author(s) reviewed and edited the content as needed and take(s) full responsibility for the content of the publication.

## Data Accessibility Statement

Data, metadata and R-codes of the analyses are reported in Open Science Framework (https://doi.org/10.17605/OSF.IO/ST7C5).

## Additional File

The additional file for this article can be found as follows:

10.5334/joc.388.s1 Supplementary Materials. The Supplementary materials contains additional analyses not reported in the paper. Specifically, it contains, separately for both experiments, analyses on prospective memory performance considering the additional external-control condition, as well as analyses on ongoing task performance and effect size sensitivity analyses. Moreover, sensitivity analyses of Bayesian models and results from pooled sample analyses are reported too.

## Footnotes

We measured OT performance too, as both accuracy and reaction times for correct trials; specifically, accuracy is a measure of the average participants’ precision at the OT, whereas the reaction times for correct trials is a readiness measure of OT stimuli’s correct detection. Moreover, reaction times can be used as a further indirect measure of monitoring, usually referred as PM cost: several studies have shown that participants performing a PM task usually are slower in the OT compared to the baseline block in which participant perform the OT without the PM task (Anderson et al., 2019); this empirical pattern reflects attentional allocation between the OT processing and the monitoring of the PM cue (Conte & McBride, 2018; Guo et al., 2019; McBride & Flaherty, 2020; Smith, 2003). However, we report all analyses on the OT performance in the Supplementary materials for completeness, because the PM cost was not the main focus of this paper.

We had also another condition in which clock-speed was not manipulated (present in both Experiment 1 and 2), but people were not allowed to check the time whenever they wished; instead, the clock appeared on the screen automatically at specific pre-set time points. This further condition, called *external-control condition*, is out of the scope of this article, and it was introduced to measure the effect of self-retrieved aspect of time monitoring, because the clock appeared on the screen automatically at specific pre-set time points, so participants did not have the possibility to check the time whenever they wished. Overall, the results from both experiments suggested that there was no effect due to self-initiated monitoring. Analyses are reported in the Supplementary materials.

The effect size of *f* = 0.33 was chosen as it corresponds to the median point between a medium effect size (*f* = 0.25) and a large effect size (*f* = 0.40), according to Cohen’s rules of thumb (Cohen, 1988, pp. 284–288).

As in Experiment 1, we included a measure for the PM cost which has not been included in the present article (see Supplementary materials for more information). However, in Experiment 2 we did not include the second OT after the TBPM tasks, because we wanted to limit the overall duration of the experimental procedure, decreasing the rate of online withdrawn by participants, as shown by previous studies (Finley & Penningroth, 2015; Logie & Maylor, 2009).
