# Supplementary material for: Keeping the Time: The Impact of External Clock-Speed Manipulation on Time-Based Prospective Memory
Source: J Cogn. 2024 Jul 16;7(1):56. doi: 10.5334/joc.388 (PMC11259119; doi:10.5334/joc.388)
Supplement: Supplementary Materials. — The Supplementary materials contains additional analyses not reported in the paper. Specifically, it contains, separately for both experiments, analyses on prospective memory performance considering the additional external-control condition, as well as analyses on ongoing task performance and effect size sensitivity analyses. Moreover, sensitivity analyses of Bayesian models and results from pooled sample analyses are reported too. [file joc-7-1-388-s1.pdf]

Keeping the time: the impact of external clock-speed manipulation on time-based prospective memory – Supplementary Materials

As mentioned in the main article, we collected additional data for another condition in which clock-speed was not manipulated during the second TBPM block, but people were not allowed to check the time whenever they wished; instead, the clock appeared on the screen automatically at specific pre-set time points. This further condition, called *external-control condition*, was not relevant for the research aims of this article, and it was introduced to measure the effect of self-retrieved aspect of time monitoring, because the clock appeared on the screen automatically at specific pre-set time points, so participants did not have the possibility to check the time whenever they wished. The external-control condition was administered in both Experiment 1 and 2. In this supplementary document, we included analyses on the external-control condition, and jointed analyses on ongoing task (OT), as well as retrospective power analysis for the ANOVAs reported in the main article (see the paper for more information about these statistical models).

OT was analyzed separately for (1) OT accuracy – computed as mean proportion of correct responses dividing the number of correct responses by the total number of OT trials – and (2) reaction times (RTs) for correct trials (in seconds). For both analyses, the between-subjects independent variable was Clock-speed (faster vs. slower vs. external-control clock), whereas the within-subjects independent variable was Block (OT before TBPM tasks vs. first TBPM block vs. second TBPM block vs. OT after TBPM tasks). These analyses allowed to investigate the PM cost (Conte & McBride, 2018; McBride & Flaherty, 2020) as well as any possible practice or fatigue effect on the OT accuracy and RTs, which were out of the scope of the main article.

Experiment 1

Methods

Participants

On top of the sample described in the main article, we collected data from a further sub-group of 40 participants that were assigned to the external-control condition (age-range: 18-35 years; M_{age} = 23.9; SD_{age} = 4.76; 30 females). Five participants reported to have a history of neurological or major psychiatric disease within the last 5 weeks (e.g.: epilepsy, depression, anxiety), or to take psychotropic drugs or others affecting the central nervous system; these participants were excluded. The final sample consisted of 35 participants (age-range: 18-35 years; M_{age} = 24.1; SD_{age} = 4.85; 25 females).

External-control condition

Participants performed two identical TBPM blocks on the computer. For both blocks, the TBPM task was to remember to press the ENTER key on the keyboard every 4 minutes; in total, five PM responses were collected for each block; during the first TBPM block, clock-speed was not manipulated (1 second = 1000 ms), whereas in the second TBPM block, clock-speed was manipulated, and participant were assigned randomly to an experimental or control condition (faster vs. slower vs. control vs. external-control; between-subject manipulation – see main articles for more information about both the faster and slower clock conditions). The clock-speed was not manipulated in the external-control condition (1 second = 1000 ms), and the external clock appeared on the screen automatically at specific pre-set time points (i.e.: 1 time during the 1st task minute; 2 times during the 2nd task minute; 3 times during the 3rd task minute; and 6 times during the 4th task minute; the last clock appearance before the PM target time occurred always 10 seconds

before its occurrence); we chose to use these specific distribution of clock appearance to resemble the "J-shaped" monitoring curve.

Results

External-control condition – analyses on prospective memory performance

Two mixed-design ANOVA were carried out separately for (1) the rate of TBPM task completion – as mean proportion of the number of PM tasks accomplished, regardless of the timing of the PM responses – and (2) the timing error of the PM responses (as difference in seconds between the actual time point when people performed the TBPM task, and the objective time point required by the TBPM task; positive values indicated later PM responses; negative values indicated earlier PM responses). For both analyses, the between-subjects independent variable was Clock-speed (faster vs. slower vs. external-control), whereas the within-subjects independent variable was Block (first TBPM block vs. second TBPM block). The detailed results can be consulted in the OSF repository (files: "LAB – external-control.jasp").

The analysis on the rate of TBPM task completion revealed no significant main effect of Block (p = 0.856) and Clock-speed (p = 0.161), and no significant interaction Block * Clock-speed (p = 0.104). The analysis on the timing error of the PM responses revealed no significant main effect of Block (p = 0.438) and no significant interaction Block * Clock-speed (p = 0.194). However, the main effect of Clock-speed was significant, $F(2, 97) = 5.02$, p = 0.008, $\omega^2_p = 0.03$, with participants exposed to faster clock having lower temporal precision in their PM responses (M = 8.28, SD = 10.96) compared to participants exposed to slower clock (M = -0.38, SD = 10.97). Overall, these results suggested that there was no effect due to self-initiated monitoring.

OT performance

Analysis on OT accuracy showed a significant main effect of Block, $F(1.42, 89.56) = 24.57$, $p < 0.001$, $\omega^2_p = 0.18$, but no effect of Clock-speed ($p = 0.152$) as well as no significant interaction Block * Clock-speed ($p = 0.211$). Post-hoc comparisons showed that OT accuracy was significantly higher for the OT performed before the TBPM tasks (i.e.: without the TBPM task; $M = 0.98$, $SD = 0.03$), compared to the OT performed during the first TBPM block ($M = 0.88$, $SD = 0.08$), $t(153) = 7.23$, $p < 0.001$, $d = 1.14$, 95% CI [0.64, 1.63], but not compared to the OT performed during the second TBPM block ($p = 1$). Moreover, people performed significantly poorly at the OT during the first TBPM block compared to the second TBPM task ($M = 0.98$, $SD = 0.02$), $t(63) = -7.60$, $p < 0.001$, $d = -1.19$, 95% CI [-1.70, -0.69], as well as compared to the OT performed after the two TBPM tasks ($M = 0.94$, $SD = 0.11$), $t(63) = -4.57$, $p < 0.001$ $d = -0.72$, 95% CI [-1.17, -0.27]. No significant difference emerged for comparison across the two OTs performed without intentions ($p = 0.050$).

RTs for incorrect trials, as well too fast (< 0.150 s) or too slow (> 3 s) responses, were excluded from the analyses. Analysis on RTs for correct OT trials revealed a main effect of the Block, $F(1.81, 114.18) = 9.48$, $p < 0.001$, $\omega^2_p = 0.06$; the main effect of Clock-speed ($p = 0.147$) as well as the interaction effect of Block * Clock-speed ($p = 0.510$) were not significant. When the OT was performed alone (i.e.: without the TBPM task), RTs did not differ between the OT performed before ($M = 0.767$, $SD = 0.109$) and after the TBPM blocks ($M = 0.742$, $SD = 0.150$; $p = 1$). Instead, people were significantly faster at the OT performed before the TBPM blocks compared to the OT performed during the first TBPM block ($M = 0.870$, $SD = 0.144$), $t(63) = -4.23$, $p < 0.001$, $d = -0.61$, 95% CI [-1.01, -0.20]. Moreover, people were significantly slower at the OT during the first TBPM block compared to the OT performed after the two TBPM tasks, $t(63) = 4.78$, $p < 0.001$, $d = 0.68$, 95% CI [0.27, 1.10].

Sensitivity analysis on effect sizes

Sensitivity analyses on the effect size can help identify the minimal effect size that the collected sample can reliably detect, considering the chosen significance level ($\alpha = 0.05$) and the predetermined power level (0.80). In Experiment 1, with group sample sizes of 27 (faster clock) and 38 (slower clock), our test and design were adequately sensitive (power > 0.8) to effect sizes of $d > 0.72$. Conversely, we likely failed to detect (power less than 50%) effect sizes smaller than $d <$ 0.50.

Experiment 2

Methods

Participants

As in Experiment 1, on top of the sample described in the main article, we collected data from a further sub-group of 40 participants that were assigned to the external-control condition (age-range: 18-35 years; $M_{age} = 27.6$; $SD_{age} = 4.88$; 18 females).

Results

External-control condition – analyses on prospective memory performance

As in Experiment 1, two mixed-design ANOVA were carried out separately for (1) the rate of TBPM task completion – as mean proportion of the number of PM tasks accomplished, regardless of the timing of the PM responses – and (2) the timing error of the PM responses (i.e.: the ratio between the time-point when people performed the TBPM task and the time-point required by the TBPM task; values above 1 indicated later PM responses; values below 1 indicated earlier PM responses). For both analyses, the between-subjects independent variable was Clock-speed (faster vs. slower vs. control vs. external-control), whereas the within-subjects independent

variable was Block (first TBPM block vs. second TBPM block). The analysis on the rate of TBPM task completion revealed no significant main effect of Block (p = 0.658) and Clock-speed (p = 0.082), and no significant interaction Block * Clock-speed (p = 0.572). The analysis on the timing error of the PM responses revealed no significant main effect of Block (p = 0.360) and Clock-speed (p = 0.716), and no significant interaction Block * Clock-speed (p = 0.088). Overall, these results confirmed that there was no effect due to self-initiated monitoring.

OT performance

We analyzed the data using mixed-design ANOVA separately for (1) OT accuracy – computed as mean proportion of correct responses dividing the number of correct responses by the total number of OT trials – and (2) reaction times (RTs) for correct trials (in seconds). For both analyses, the between-subjects independent variable was Clock-speed (faster vs. slower vs. control vs. external-control condition), whereas the within-subjects independent variable was Block (OT before TBPM tasks vs. first TBPM block vs. second TBPM block). Analysis on OT accuracy showed a significant main effect of Block, $F(1.71, 191.28) = 14.10$, $p < 0.001$, $\omega^2_p = 0.03$; the main effect of Clock-speed (p = 0.090) as well as the interaction Block * Clock-speed (p = 0.080) were not significant. Post-hoc comparisons showed that OT accuracy was significantly higher for the OT alone (i.e.: without the TBPM task; M = 0.968, SD = 0.031), compared to the OT performed during the first TBPM block (M = 0.957, SD = 0.030), $t(112)$ = 4.47, p < 0.001, d = 0.34, 95% CI [0.15, -0.53], as well as compared to the OT performed during the second TBPM block (M = 0.956, SD = 0.031), $t(112)$ = 4.72, p < 0.001, d = 0.36, 95% CI [0.17, 0.55]. No significant difference was found between the two TBPM blocks (p = 1).

As in Experiment 1, RTs for incorrect trials, as well too fast (< 0.150 s) or too slow (> 3 s) responses, were excluded from the analyses. Analysis on RTs for correct OT trials showed a

significant main effect of Block, $F(1.46, 163.60) = 4.20$, $p = 0.027$, $\omega^2_p = 0.01$; the main effect of Clock-speed ($p = 0.475$) and the interaction Block * Clock-speed ($p = 0.385$) were not significant. Post-hoc comparisons showed that RTs for correct OT trials were significantly faster for the OT alone (i.e.: without the TBPM task; *M* = .823, *SD* = .203), compared to the OT performed during the first TBPM block (*M* = .860, *SD* = .172), $t(112) = -2.78$, $p = 0.003$, $d = -0.19$, 95% CI [-0.35, -0.02], but not compared to the OT performed during the second TBPM block (*M* = .845, *SD* = .160; $p = 1$).

Sensitivity analysis on effect sizes

As in Experiment 1, sensitivity analysis on the effect size was carried out to identify the minimal effect size that the collected sample can reliably detect, considering the chosen significance level ($\alpha = 0.05$) and the predetermined power level (0.80). In Experiment 2, with group sample sizes of 36 (faster clock) and 39 (slower clock or control condition), our test and design were adequately sensitive (power > 0.8) to effect sizes of $d > 0.66$. Conversely, we likely failed to detect (power less than 50%) effect sizes smaller than $d < 0.46$. With group sample sizes of 39 (slower clock vs. control condition), our test and design were adequately sensitive (power > 0.8) to effect sizes of $d > 0.64$. Conversely, we likely failed to detect (power less than 50%) effect sizes smaller than $d < 0.45$.

Sensitivity analyses of Bayesian models

Sensitivity analyses were carried out on Bayesian models to test the robustness of the results (Depaoli et al., 2020). The aim was to reproduce analyses on performed in the main paper using three different priors for the fixed effects: informative (0.5; the default option in JASP), weakly informative (0.707), and diffuse (1). The detailed results can be consulted in the OSF repository

(files: “LAB – Bayes sensitivity.jasp” and “ONLINE – Bayes sensitivity.jasp”; https://doi.org/10.17605/OSF.IO/ST7C5).

Rate of TBPM Task Completion

Bayesian repeated measures ANOVAs were performed to examine the effects of Block and Clock-speed on the rate of TBPM task completion. The results of Experiment 1 consistently showed that the model including the interaction effect (Block * Clock-speed) was preferred over the null model. However, the Bayes factors ranged from 1.51 to 2.18, indicating anecdotal evidence in favor of the interaction model. The estimated R-squared values ranged from 0.159 to 0.163, indicating that the model explained approximately 16% of the variance in the rate of TBPM task completion. The results of Experiment 2 showed that the null model was preferred over the model including the interaction effect (Block * Exp.Condition). The Bayes factors ranged from 0.043 to 0.134, indicating moderate evidence in favor of the null hypothesis. The estimated R-squared values ranged from 0.086 to 0.089, indicating that the model explained approximately 9% of the variance in the rate of TBPM task completion.

Timing Error of PM Response

Bayesian repeated measures ANOVAs were conducted to investigate the effects of Block and Clock-speed on the timing error of PM response. The results of Experiment 1 indicated that the model including the interaction effect (Block * Clock-speed) was preferred over the null model for the priors 0.5 and 0.707, with Bayes factor of 1.33 and 1.12, respectively. However, for the diffuse prior (1), the null model was preferred with a Bayes factor of 0.87. The estimated R-squared values ranged from 0.291 to 0.295, suggesting that the model explained approximately 29% of the variance in the timing error of PM response. The results of Experiment 2 were consistent, indicating that the model including the interaction effect (Block * Clock-speed) was preferred over

the null model for the prior 0.5, but only with a Bayes factor of 1.28. For both the weakly informative and diffuse priors, the null model was preferred with Bayes factor of 1.08 and 0.63, respectively. The estimated R-squared values were consistent across the priors (0.169), suggesting that the model explained approximately 17% of the variance in the timing error of PM response.

Time Monitoring

Bayesian repeated measures ANOVAs were performed to examine the effects of Block, Clock-speed, and Time on time monitoring. The results of Experiment 1 consistently showed that the model including both the interaction effect (Block * Clock-speed) and the three-way interaction effect (Time * Block * Clock-speed) was preferred over the null model and the model with only the interaction effect. The Bayes factor for the preferred model ranged from 11.35 to 234.85, indicating strong to very strong evidence in favor of this model. The estimated R-squared values were consistently high (0.79), suggesting that the model explained approximately 79% of the variance in time monitoring. Consistently, the results of Experiment 2 showed that the model including both the interaction effect (Block * Clock-speed) and the three-way interaction effect (Time * Block * Clock-speed) was preferred over the null model and the model with only the interaction effect. The Bayes factor for the preferred model ranged from 8801.82 to 56603.59, indicating extremely strong evidence in favor of this model. The estimated R-squared values were consistently high (0.76), suggesting that the model explained approximately 76% of the variance in time monitoring.

Pooled sample analyses

In order to further check the consistency of the results across the experiments as well as the individual differences associated with the setting of testing (i.e., Experiment 1 was assessed in the laboratory with trained experimenters, while Experiment 2 was carried out online at participant's

home), we exploratorily re-ran all the analyses reported in the article pooling together the two samples from Experiment 1 and 2, controlling for the setting of the assessment and removing the control condition because it was present only in Experiment 2.

Results

We applied mixed-design ANOVAs with post-hoc *t*-tests corrected using multiple comparisons method for the *p*-values of the comparisons. We focused on two effects of interests, as well as on the respective post-hoc comparisons, regardless of the level of significance:

1. The interaction effect Block * Clock-speed (present in all ANOVAs), as a measure of the effect of clock-speed on the dependent variables.
2. The interaction effect Time * Block * Clock-speed, as a measure of the effect of clock-speed on the strategicness of time monitoring (this effect was present only in the analysis on time monitoring).

Descriptive statistics, data, metadata and of these analyses are reported in Open Science Framework (file: "JOINTED – results.jasp"; https://doi.org/10.17605/OSF.IO/ST7C5).

[Table 2 about here]

TBPM performance

Two mixed-design ANOVA were carried out separately for the rate of TBPM task completion, and for the timing error of the PM responses. For both analyses, the between-subjects independent variable was Clock-speed (faster vs. slower), whereas the within-subjects independent variable was Block (first TBPM block vs. second TBPM block); the Assessment (laboratory vs. online) was included as covariate.

The analysis on the rate of TBPM task completion revealed only a main effect of Assessment, $F(1, 138) = 16.33$, $p < 0.001$, $\omega^2_p = 0.06$, with participant assessed online performing

better (*M* = 0.97, *SD* = 0.12) than participants assessed in the laboratory (*M* = 0.90, *SD* = 0.142). The analysis on the timing error of the PM responses did not reveal any significant main effect of Block (p = 0.294) and Assessment (p = 0.517), as well as no interaction effect Block * Assessment (p = 0.202); however, a main effect of Clock-speed was found, $F(1, 138) = 9.48$, $p = 0.003$, ω^2_p = 0.03, as well as a significant interaction effect Block * Clock-speed, $F(1, 138) = 1.64$, $p = 0.010$, ω^2_p = 0.02. The difference in the response's deviations between the faster and slower clock conditions was not found selectively in the second TBPM block, with later PM responses in the faster clock condition (*M* = 6.91, *SD* = 13.02) compared to slower clock condition (*M* = 0.96, *SD* = 11.66), $t(139) = 4.01$, $p < 0.001$, $d = 0.68$, 95% CI [0.23, 1.37]; the mean response's deviation from the PM target time did not differ significantly among each other during the first TBPM block (p = 1). Bayesian analysis was carried out testing the alternative model comprising the interaction effect of interest Block * Clock-speed against a null model containing the main effects of Clock-speed, Block and Participants; the Bayes Factor was 4.52, indicating moderate evidence for the alternative hypothesis (Wetzels et al., 2015).

In addition, we ran a series of one-sample *t*-tests to furtherly assess whether the timing of the PM responses in each condition were significantly different from zero, which represented PM responses with no timing error. The analysis on the PM responses during the first TBPM task block for participants exposed later to the faster clock indicated that participants were significantly off-time, $t(63) = 2.69$, $p = 0.009$, $d = 0.34$, 95% CI [0.08, 0.59]; however, the Bayes Factor was 3.60, indicating only moderate evidence. The analysis on the PM responses during the second TBPM task block for participants exposed to the faster clock indicated that participants were significantly off-time, $t(63) = 4.25$, $p < 0.001$, $d = 0.53$, 95% CI [0.27, 0.69]; the Bayes Factor was > 30, indicating very strong evidence. The *t*-test on the PM responses during both TBPM task blocks for participants exposed to the slower clock indicated that participants were on-time during the second

TBPM block (p = 0.471), but not during the first TBPM block, t(77) = 2.27, p = 0.026, d = 0.26, 95% CI [0.03, 0.49]; the Bayes Factor was 1.41, indicating only anecdotal evidence for the alternative hypothesis.

Time monitoring

A mixed-design ANOVA was carried out to measure the effect of Clock-speed (faster vs. slower clock) as between-subject variable, and Block (first TBPM block vs. second TBPM block) and Time (minute 1 vs. minute 2 vs. minute 3 vs. minute 4) as within-subject variables, on time monitoring (measured as mean clock check frequency per minute); the Assessment (laboratory vs. online) was included as covariate. The statistical analysis showed a main effect of Assessment, $F(1, 138) = 40.12$, $p < 0.001$, $\omega^2_p = 0.12$, and Time, $F(1.62, 223.63) = 67.64$, $p < 0.001$, $\omega^2_p = 0.10$, as well as interaction effects of Time * Assessment, $F(1.62, 223.63) = 37.54$, $p < 0.001$, $\omega^2_p = 0.06$, Block * Clock-speed, $F(1, 138) = 9.72$, $p = 0.002$, $\omega^2_p = 0.01$, and Time * Block * Clock-speed, $F(2.87, 395.87) = 17.53$, $p < 0.001$, $\omega^2_p = 0.01$.

Post-hoc analyses for the main effect of Time revealed that people checked the clock strategically overall; specifically, the results showed that participants checked the clock less frequently in minute 1 (M = 0.55, SD = 1.03) compared to minute 2 (M = 0.87, SD = 1.37), t(63) = -4.90, p < .001, d = -0.23, 95% CI [-0.44, -0.03], minute 3 (M = 1.09, SD = 1.34), t(63) = -8.54, p < .001, d = -0.39, 95% CI [-0.60, -0.17], and minute 4 (M = 2.38, SD = 1.76), t(63) = -12.25, p < .001, d = -1.30, 95% CI [-1.67, -0.93]. Similarly, participants checked the clock less in minute 2 compared to minute 3, t(63) = -5.03, p < .001, d = -0.15, 95% CI [-0.35, -0.05], and minute 4, t(63) = -11.83, p < .001, d = -1.07, 95% CI [-1.40, -0.75]. Clock check frequency was significantly lower in minute 3 than minute 4 too, t(63) = -11.11, p < .001, d = -0.92, 95% CI [-1.22, -0.62].

Post-hoc comparisons for the interaction effect Block * Clock-speed showed that people exposed to the slower clock increased significantly clock checks frequency from the first TBPM block (*M* = 1.29, *SD* = 0.03) to the second TBPM block (*M* = 1.60, *SD* = 0.04), $t(63) = -3.23$, $p = 0.011$, $d = -0.22$, 95% CI [-0.40, -0.03]. The same comparisons for participants in the faster clock condition did not show significant results ($p > 0.013$). Post-hoc comparisons for the interaction effect Time * Block * Clock-speed furtherly showed that the interaction effect Block * Clock-speed occurred only during the third and fourth minute before the PM target time; specifically, people exposed to the slower clock increased significantly clock check frequency from the first TBPM block to the second TBPM block, but only on minute 4 ($M_{first\ TBPM\ block} = 2.34$, $SD_{first\ TBPM\ block} = 1.74$; $M_{second\ TBPM\ block} = 2.92$, $SD_{second\ TBPM\ block} = 2.22$), $t(63) = -3.63$, $p < 0.001$, $d = -0.42$, 95% CI [-0.76, -0.08]. The same comparisons for minute 1 and 2, as well as the comparison with the faster clock condition, did not show significant results ($p > 0.003$). Bayesian analysis was carried out testing the alternative model comprising the interaction effects of interest (i.e., Block * Clock-speed, and Time * Block * Clock-speed) against a null model containing the main effects of Clock-speed, Block and Participants, as well as the interaction effects of Block * Time, and Time * Clock-speed; the Bayes Factor was 23.46 for the effect Block * Clock-speed, indicating strong evidence for the alternative hypothesis, and > 30 for the effect Time * Block * Clock-speed, indicating very strong evidence for the alternative hypothesis (Wetzels et al., 2015).

Post-hoc analyses for the main effect of Assessment revealed significant differences across samples in the total amount of clock checks; specifically, the results showed that participants each minute checked the clock less frequently in the laboratory (*M* = 1.26, *SD* = 1.38) compared to online setting (*M* = 2.51, *SD* = 1.58). The significant interaction effect Time * Assessment further confirmed lower clock check in the laboratory compared to online assessment consistently over time ($p < 0.001$).

Discussion

Overall, results suggested that the effects of interested were consistent across experiments. Specifically, it emerged consistently that clock-speed did not affect PM task completion, but only the timing of the response during the second TBPM block, with participants in the slower clock condition performing earlier PM responses compared to participants in the faster clock condition; moreover, the main effect of clock-speed was significant as well. Regarding time monitoring, the results confirmed a within-participants increase of clock check across TBPM blocks at minute 4 for participants in the slower clock condition, and a significant difference between participants in the faster and slower clock, always at minute 4 of the second TBPM block.

References

Conte, A. M., & McBride, D. M. (2018). Comparing time-based and event-based prospective memory over short delays. *Memory*, *26*(7), 936–945. https://doi.org/10.1080/09658211.2018.1432662

Depaoli, S., Winter, S. D., & Visser, M. (2020). The Importance of Prior Sensitivity Analysis in Bayesian Statistics: Demonstrations Using an Interactive Shiny App. *Frontiers in Psychology*, *11*. https://doi.org/10.3389/fpsyg.2020.608045

McBride, D. M., & Flaherty, M. (2020). Comparing costs in time-based and event-based prospective memory. *Memory*, *28*(7), 918–925. https://doi.org/10.1080/09658211.2020.1798463

Wetzels, R., Van Ravenzwaaij, D., & Wagenmakers, E.-J. (2015). Bayesian Analysis. In R. L. Cautin & S. O. Lilienfeld (Eds.), *The Encyclopedia of Clinical Psychology* (pp. 1–11). John Wiley & Sons, Inc. https://doi.org/10.1002/9781118625392.wbecp453
